# Case Report: Right-atrial reverse remodeling causing resolution of new-onset torrential tricuspid regurgitation after successful rhythm control

**DOI:** 10.3389/fcvm.2026.1762249

**Published:** 2026-04-10

**Authors:** Grigorios Korosoglou, Wolfgang Fehske, Patrick Lugenbiel, Norbert Frey

**Affiliations:** 1 Department of Cardiology and Vascular Medicine, GRN Hospital Weinheim, Weinheim, Germany; 2 Cardiac Imaging Center Weinheim, Hector Foundation, Weinheim, Germany; 3 Department of Cardiology, Heart Center, University Hospital Cologne, Cologne, Germany; 4 Department of Cardiology, Angiology, and Pneumology, University Hospital of Heidelberg, Heidelberg, Germany; 5 DZHK (German Centre for Cardiovascular Research), Partner Site Heidelberg/Mannheim, Heidelberg, Germany

**Keywords:** atrial cardiomyopathy, atrial fibrillation, atrial remodeling, atrial secondary tricuspid regurgitation, pacemaker lead, tricuspid clipping

## Abstract

In patients with persistent atrial fibrillation, adverse atrial remodeling may induce annular enlargement causing severe mitral- or tricuspid valve regurgitation and heart failure symptoms. Herein, we describe a case of rapidly progressive right atrial (RA) adverse remodeling due to new onset persistent AF, causing tricuspid annular dilatation and torrential tricuspid regurgitation (TR) in a 79-year-old female patient. This condition could be diagnosed by echocardiography, which helped guiding further patient management. After successful electric cardioversion, reverse RA remodeling took place, resulting in resolution of RA enlargement, TR and clinical symptoms within a few weeks. To maintain sinus rhythm, the patient underwent cryoablation and her further clinical course was uneventful.

## Introduction

Atrial fibrillation (AF) is one major risk factor for heart failure development and progression in the general population. Based on previous studies, restoration and maintenance of sinus rhythm (SR) may be associated with improved cardiovascular mortality and reduced rates of stroke compared to a pure heart rate control strategy (1, 2). In patients, where SR cannot be restored and AF persists, on the other hand, atrial myopathy may occur, causing eventually irreversible adverse atrial remodeling. Additional cardiovascular comorbidities, like arterial hypertension and/or coronary artery and valvular heart disease if present, may promote adverse remodeling, ultimately resulting in compromised ventricular function and clinically evident heart failure (1).

In the recent EAST-AFNET 4 (Early Rhythm-Control Therapy in Patients With Atrial Fibrillation) trial, which randomly assigned patients with early AF to rhythm control vs. usual care, rhythm control was associated with a lower risk for adverse cardiovascular outcomes, including cardiac death, stroke and hospitalization due to heart failure or acute coronary syndrome, compared to usual care. Notably, rhythm control included both treatment with antiarrhythmic drugs (45.7% still receiving antiarrhythmic drugs after 2 years) or AF catheter ablation (19.4%). However, symptoms and left ventricular (LV)-function at 2 years did not differ significantly between the groups so that the exact pathophysiological mechanism causing improved outcomes is not yet fully understood.

Imaging modalities such as echocardiography and if required cardiac magnetic resonance (CMR), combined with invasive electro-anatomical mapping, could provide valuable information for the characterization of electrical, contractile and structural atrial substrate, guiding optimal patient management. Herein, we present a patient with new-onset persistent atrial fibrillation, which rapidly caused right atrial remodeling and severe heart failure symptoms. The role of cardiac imaging by echocardiography was decisive for identifying the etiology of heart failure symptoms and for guiding patient management.

## Case description

A 79-year-old female patient was referred for routine pacemaker control in our department. The patient had undergone implantation of a dual-chamber pacemaker (Medtronic Ensura DR MRI) 7 years ago due to syncopal episodes and had a history of paroxysmal AF, which was so far asymptomatic. In addition, she had history of mild coronary artery disease without high-grade lesions by cardiac computed tomography angiography (CAD RADS 2 with 25%–50% stenosis 3 years ago) and mild plaque formation in her carotid arteries without hemodynamically significant stenosis by Duplex ultrasonography. The patient also had a history of arterial hypertension, treated with 5 mg Ramipril and hyperlipidemia, treated with 20 mg atorvastatin. In addition, she was on oral anticoagulation with rivaroxaban 15 mg daily (CHA_2_DS_2_-VA-Score of 3). During the index presentation, she reported on new-onset heart failure symptoms with exertional dyspnea NYHA class III during the last 2 weeks.

**Figure 1:**
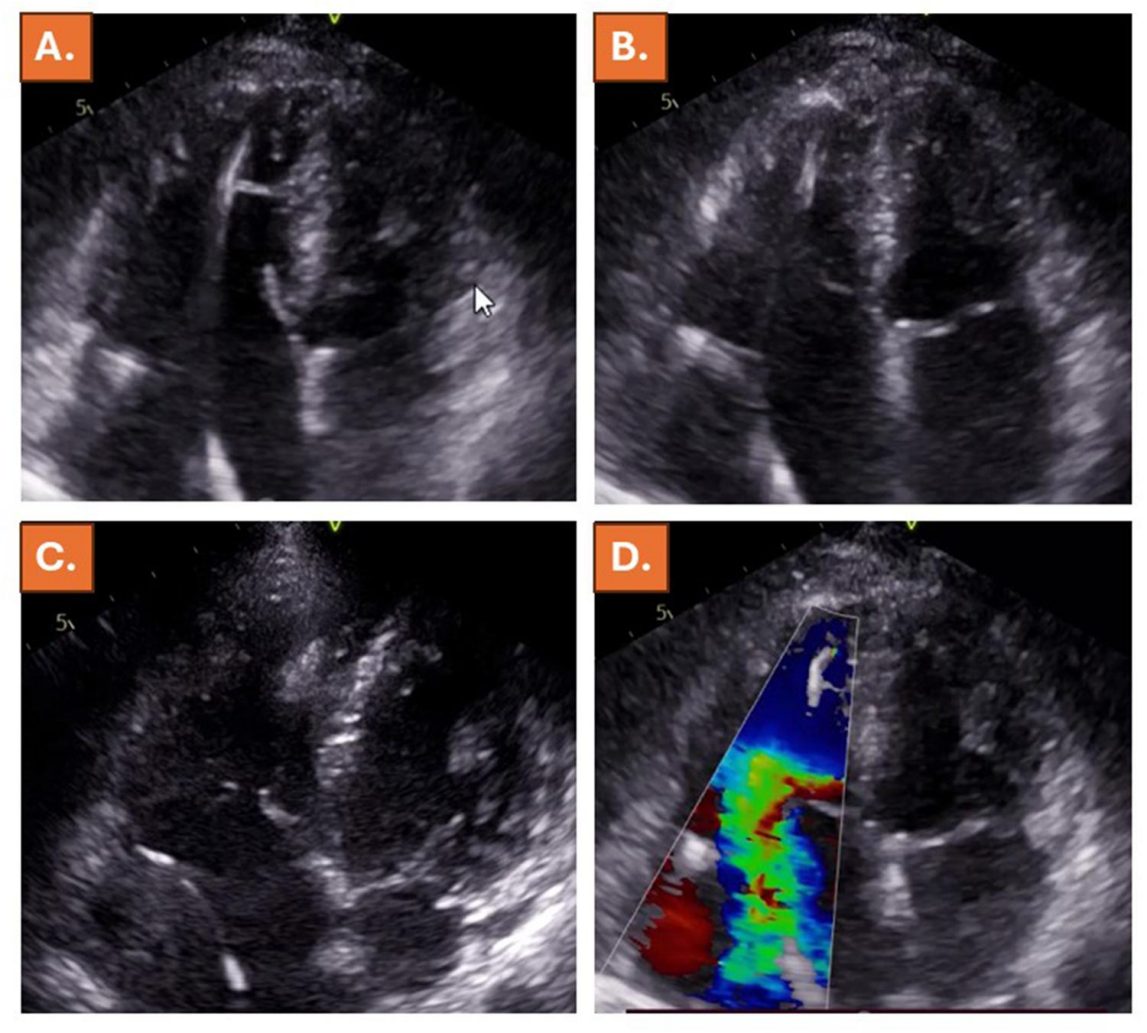
Normal diameters and function of the LV and LA but enlargement of the RV, reduced RV-function and severe RA enlargement by echocardiography. Systolic image in **(A)**, diastolic image in **(B)**, coaptation gap of 6 mm in **(C)** and torrential TR by Doppler ultrasound shown in **(D)**.

By physical examination an elevated heart rate (90 bpm) with arrhythmia was noted. ECG revealed atrial fibrillation with 90 bpm without ischemic signs. Based on pacemaker interrogation, at that time, persistent atrial fibrillation was noted during the last 18 days. Echocardiography was performed due to new-onset heart failure symptoms, revealing normal diameters and function of the left ventricle (LV) and of the left atrium (LA) (LV-end-diastolic diameter of 41 mm, LA diameter of 38 mm and LA-area of 14.8 cm^2^) but enlargement of the right ventricle (RV) (end-diastolic diameter of 46 mm), mildly reduced RV-function (ejection fraction of 48%) and right atrial RA enlargement (RA diameter of 47 mm, RA area of 22.0 cm^2^) (Table 1, Figures 1A, B, Supplementary Video 1). In addition, Doppler sonography revealed torrential tricuspid regurgitation (TR) caused by a coaptation gap of 6 mm and tricuspid annular dilatation (Figures 1C, D, Supplementary Video 2).

**Table 1:** Overview of the echocardiographic parameters over time.

Timepoints	6 months prior to the index presentation	Index presentation	2 weeks after the index presentation	6 weeks after the index presentation	6 months after the index presentation
LV diameter (mm)	42	41	44	42	40
RV diameter (mm)	37	46	47	38	38
LVEF (%)	56	54	55	54	54
RVEF (%)	52	48	37	55	54
LA diameter (mm) (septal-lateral)	36	38	36	37	36
LA area (cm^2^)	13.8	14.8	14.2	14.0	13.6
RA diameter (mm) (septal-lateral)	39	47	55	42	44
RA area (cm^2^)	16.3	22.0	25.0	16.6	17.9
NT pro BNP (ng/L)	293	1,125	1,446	Not available	363

RV, right ventricle; LV, left ventricle; LVEF, left ventricular ejection fraction; RVEF, right ventricular ejection fraction; LA, left atrium; RA, right atrium.

**Figure 2:**
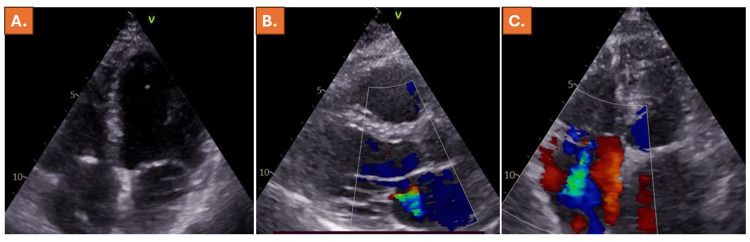
Normal LV-, RV, LA and RA diameters **(A, B)** and only mild TR in **(C)**, six months prior to the index presentation of the patient.

**Figure 3:**
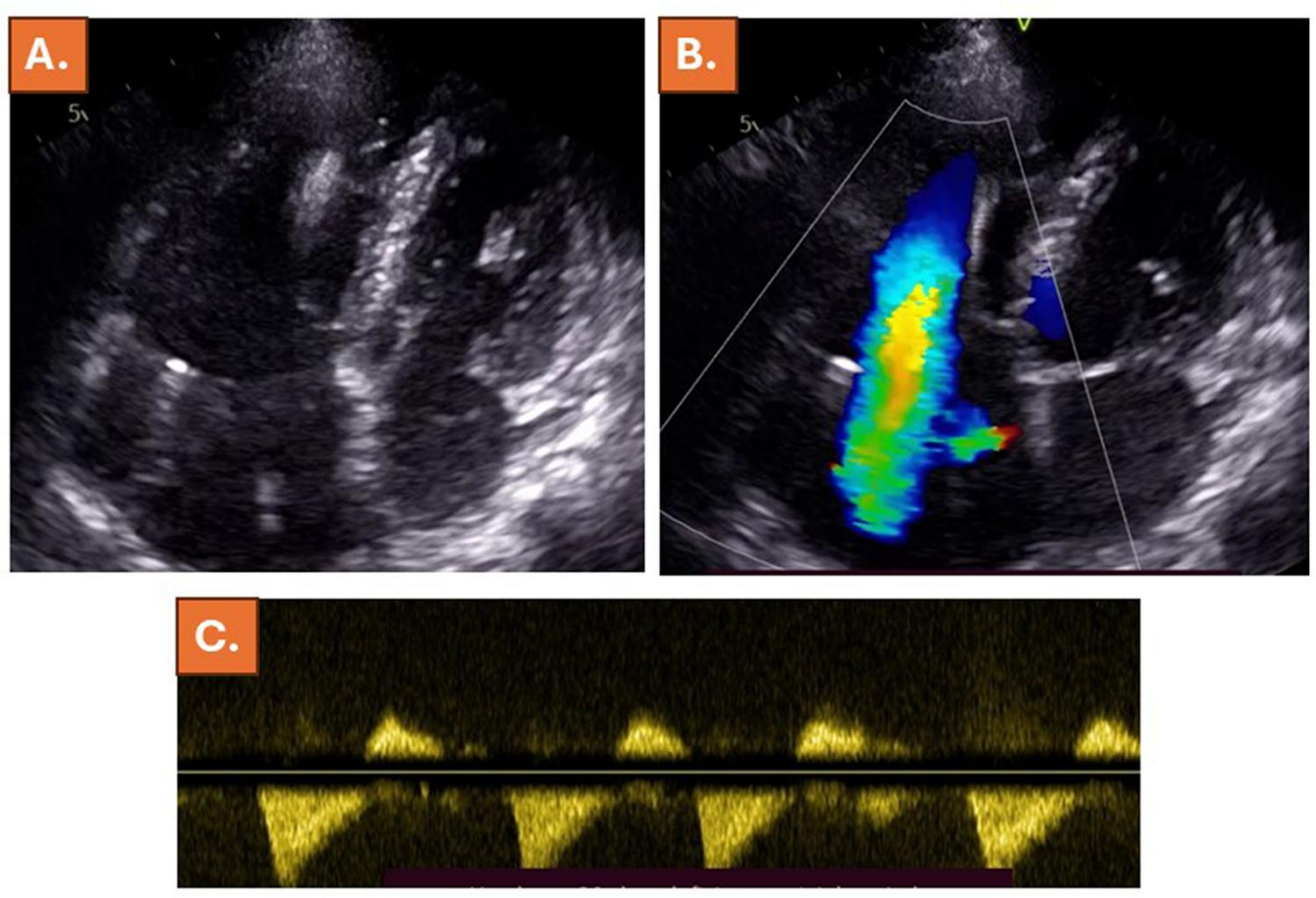
Echocardiography 2 weeks after the index presentation showed further worsening of the RV-function, accompanied by increased dilatation of the RV and D-shape of the LV **(A)**, persistent torrential TR **(B)** with a triangular TR shape **(C)**.

Review of echocardiographic images, which were acquired during routine echocardiography 6 months prior to the index presentation of the patient revealed normal LV-, RV- and RA-diameters and volumes (Table 1) and only mild TR (Figures 2A–C, Supplementary Videos 3–5). Since the patient had undergone dual-chamber pacemaker implantation 7 years ago, the presence of pacemaker lead associated TR was considered unlikely, and torrential atrial secondary tricuspid regurgitation (ASTR) was suspected due to new-onset AF. Due to onset heart failure symptoms, the patient was set on additional treatment with 10 mg bisoprolol, 25 mg eplerenone and 10 mg dapagliflozin per day. In addition, ramipril was exchanged with sacubitril-valsartan 24/26 mg twice per day, 5 mg of the loop diuretic torsemide were administrated due to congestion symptoms, and the patient was scheduled for electric cardioversion after 2 weeks. Echocardiography 2 weeks after index presentation exhibited further worsening of the RV-function, accompanied by increased dilatation of the RV and D-shape of the LV (Table 1), persistent torrential TR with a triangular regurgitation shape and congestion with lack of respiratory variability of the inferior vena cava (Figures 3A–C, Supplementary Videos 6–8). In addition, NT-pro BNP significantly increased (from 293 ng/L prior to the index presentation to 1,125 ng/L during the index presentation, further rising to 1,446 ng/L 2 weeks after the index presentation of the patient, Table 1). Electric cardioversion was successfully performed, and the patient was discharged on the same day.

**Figure 4:**
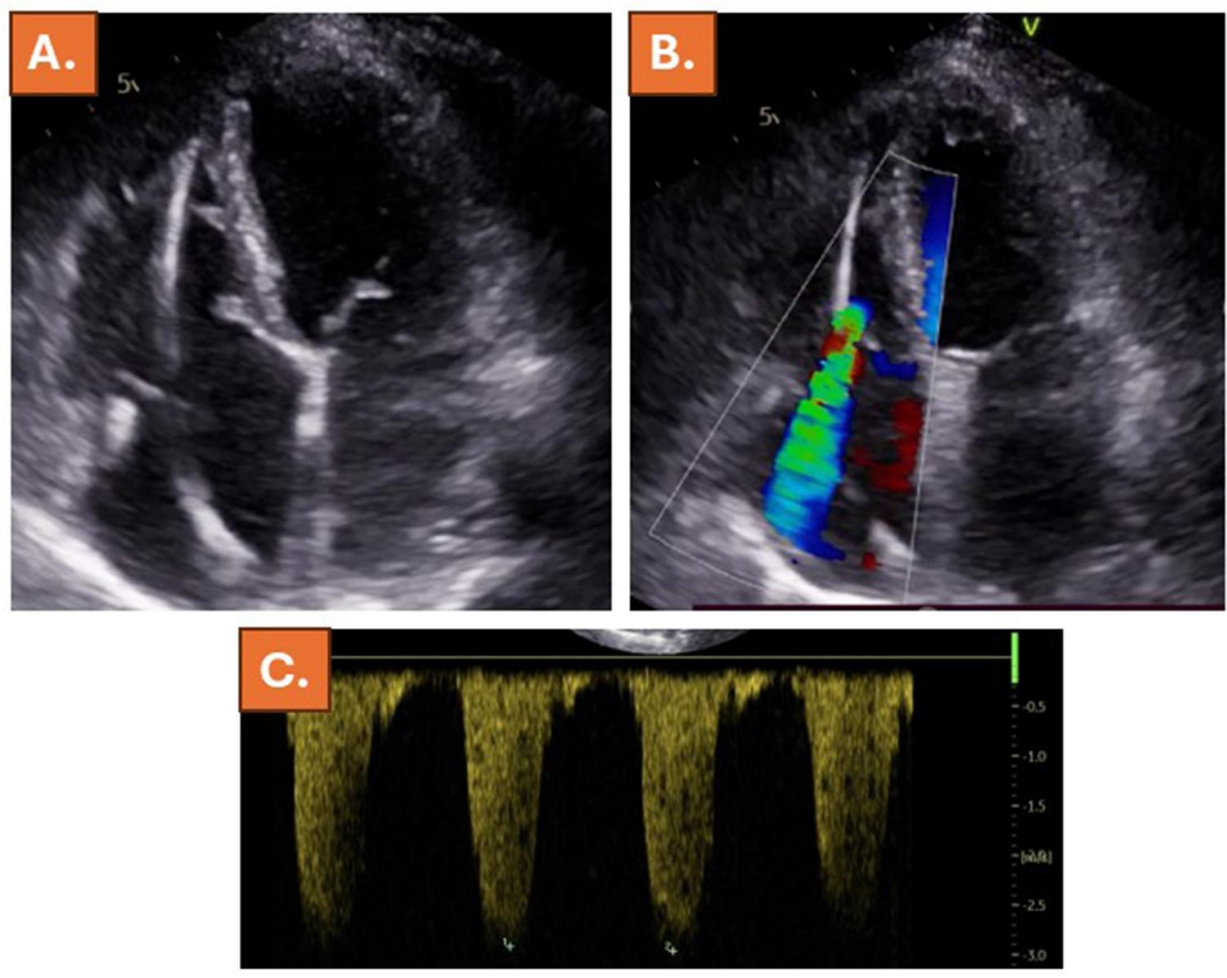
Four weeks after successful electric cardioversion, echocardiography revealed normalization of RV-diameters and function **(A)** and only mild- to moderate tricuspid regurgitation **(B)** with slightly elevated systolic pulmonary artery pressure of 40mmHg **(C)**.

The patient was rescheduled for an ambulatory clinical visit after 4 weeks (6 weeks after the index presentation), where she reported an almost complete resolution of exertional dyspnea (NYHA class I-II). Echocardiography revealed normalization of RV-diameters and function and only mild- to moderate tricuspid regurgitation (Table 1) with slightly elevated systolic pulmonary artery pressure (40 mmHg), whereas no signs of congestion were present (Figures 4A–C, Supplementary Videos 9–11). After 2 months, pulmonary vein cryoablation was performed. During the following ambulatory control visits, the patient remained on stable sinus rhythm with complete resolution of heart failure symptoms, returning to a physically active lifestyle. Echocardiography after 6 months revealed completely normal findings, including normalized RV-diameters and function, mild tricuspid regurgitation and no signs of congestion (Table 1, Supplementary Videos 12–14). In addition, NT-pro BNP was within normal range (363 ng/L).

## Discussion

AF is the most frequent arrhythmia detected in daily clinical practice, being associated with impaired quality of life, reduced life expectancy, and socioeconomic costs (3). With AF, both electric and structural atrial remodeling seem to play a pivotal role as contributors of the arrhythmogenic substrate. In this regard, fibrosis, induced by accumulation of fibrillar collagen deposits, causes interstitial expansion, which is a hallmark of the arrhythmogenic structural remodeling (4). With persistence of such processes, irreversible structural remodeling, so called ‘atrial myopathy’ may occur, which may prevent conversion or/and maintenance of SR despite electric cardioversion, ablation procedures, or/and antiarrhythmic treatment and forecast heart failure and other cardiovascular complications due to AF (5). Our case describes a case of ASTR, which occurs most commonly in elderly women with atrial fibrillation and in heart failure with preserved ejection fraction in sinus rhythm (6). In ASTR, leaflet malcoaptation mainly results from significant tricuspid annulus dilation due to right atrial enlargement. This condition represents 10%–15% of clinically relevant tricuspid regurgitation and generally has better outcomes than the ventricular type of TR. Recent studies indicate that patients with ASTR may benefit from aggressive rhythm control, like in the patient described herein (6).

Echocardiography is the most widespread, practical and cost-effective imaging modality, playing a pivotal role for the evaluation of patients with AF and in ASTR. Thus, echocardiography provides valuable information on cardiac and valvular function, detecting LA or RA chamber dilation, spherical deformation, and reduced atrial function. LA enlargement was proposed as the first detectable anatomical change prior to the onset of AF (7), and measures of LA diameters, areas and volumes by echocardiography have been described as robust predictors of AF recurrence after electrical cardioversion or ablation (8). However, most of the previous studies focused on LA parameters for the description of atrial remodeling processes, whereas limited information is available on the role of the RA in patients with AF (9, 10).

Advanced cardiovascular imaging techniques, like CMR, on the other hand, have been proposed for the estimation of atrial fibrosis using late gadolinium enhancement techniques (11), which have been traditionally used for the assessment of myocardial viability (12). Thus, in the DECAAF I study, in patients with AF undergoing catheter ablation, atrial tissue fibrosis quantified by CMR was independently associated with likelihood of recurrent arrhythmia (11). However, in the DECAAF II trial, which also used CMR for the quantification of left atrial fibrosis, CMR-guided ablation did not result in differences in sinus rhythm maintenance compared to conventional catheter ablation (13). The authors concluded that the DECAAF II findings do not support the use of CMR-guided ablation in patients with persistent AF and since then, the assessment of left-atrial fibrosis by CMR has been largely abandoned in clinical trials and in daily practice. CMR however, can as a technique offer far more than the assessment of LA fibrosis since its tomographic nature, allows the precise and reproducible assessment of cardiac dimensions and function, irrespective of the echogenic windows of the patients (12).

The present case highlights that the nature of heart valve lesions can be dynamic, improving without requiring surgical or percutaneous interventions and the role of echocardiography for detecting abnormalities in RA, RV dimensions and function and for understanding the underlying pathophysiology in patients with ASTR. Similar cases, where rhythm control, resulted in significant ASTR improvement have been reported before, stressing the importance of early rhythm management (14). In contrast to this case, however, in our patient the presence of pacemakers lead in the RV may have led to the wrong diagnosis of a pacemaker lead associated torrential TR, which may have resulted in wrong therapeutic decisions for the patient. Echocardiography at baseline and consideration of echocardiographic images prior to the index presentation of the patient helped to exclude this diagnosis, making the scenario of an ASTR most probable. After electric cardioversion reverse atrial remodeling occurred much faster compared to the previously reported case (14), where reserve atrial remodeling was noted at 4 months of follow-up. This highlights that dynamic changes in valve disorders may strongly vary, possibly based on the extent of preexisting atrial fibrosis.

## Conclusion

Our case report underlines the functional character of atrio-ventricular valve disorders and the role of echocardiography for the assessment of TR extent and etiology. In addition, the present case highlights the role of AF as a cause of rapidly progressive ASTR due to adverse remodeling and the role of rhythm control for reversing atrial remodeling, TR and associated clinical symptoms.

## Data Availability

The raw data supporting the conclusions of this article will be made available by the authors, upon reasonable request.
