# Supplementary material for: Case Report: Right-atrial reverse remodeling causing resolution of new-onset torrential tricuspid regurgitation after successful rhythm control
Source: Front Cardiovasc Med. 2026 Apr 10;13:1762249. doi: 10.3389/fcvm.2026.1762249 (PMC13105964; doi:10.3389/fcvm.2026.1762249)
Supplement: Supplementary file 1 [file Presentation1.pptx]

## Slide 1
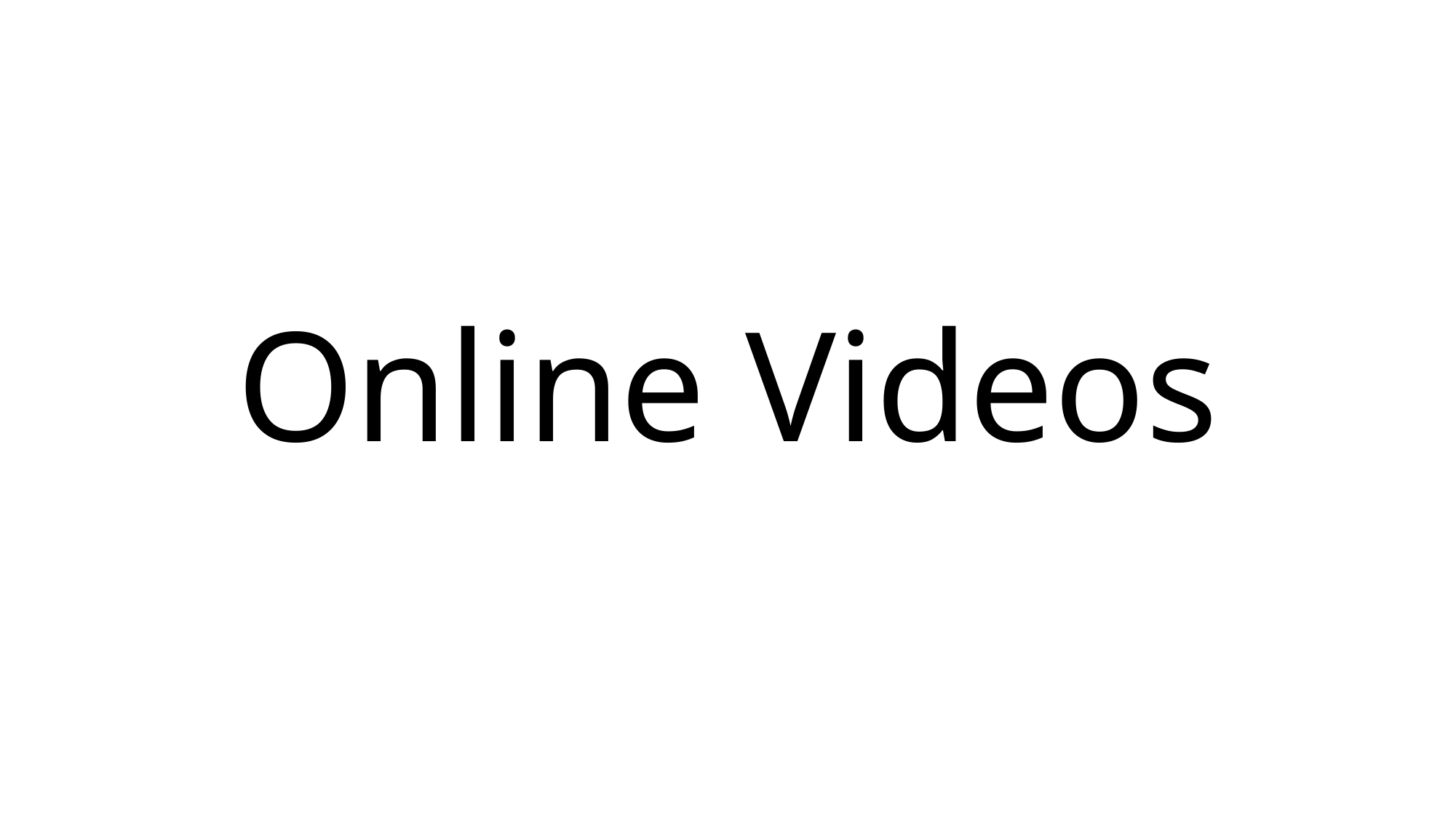

Online Videos

## Slide 2
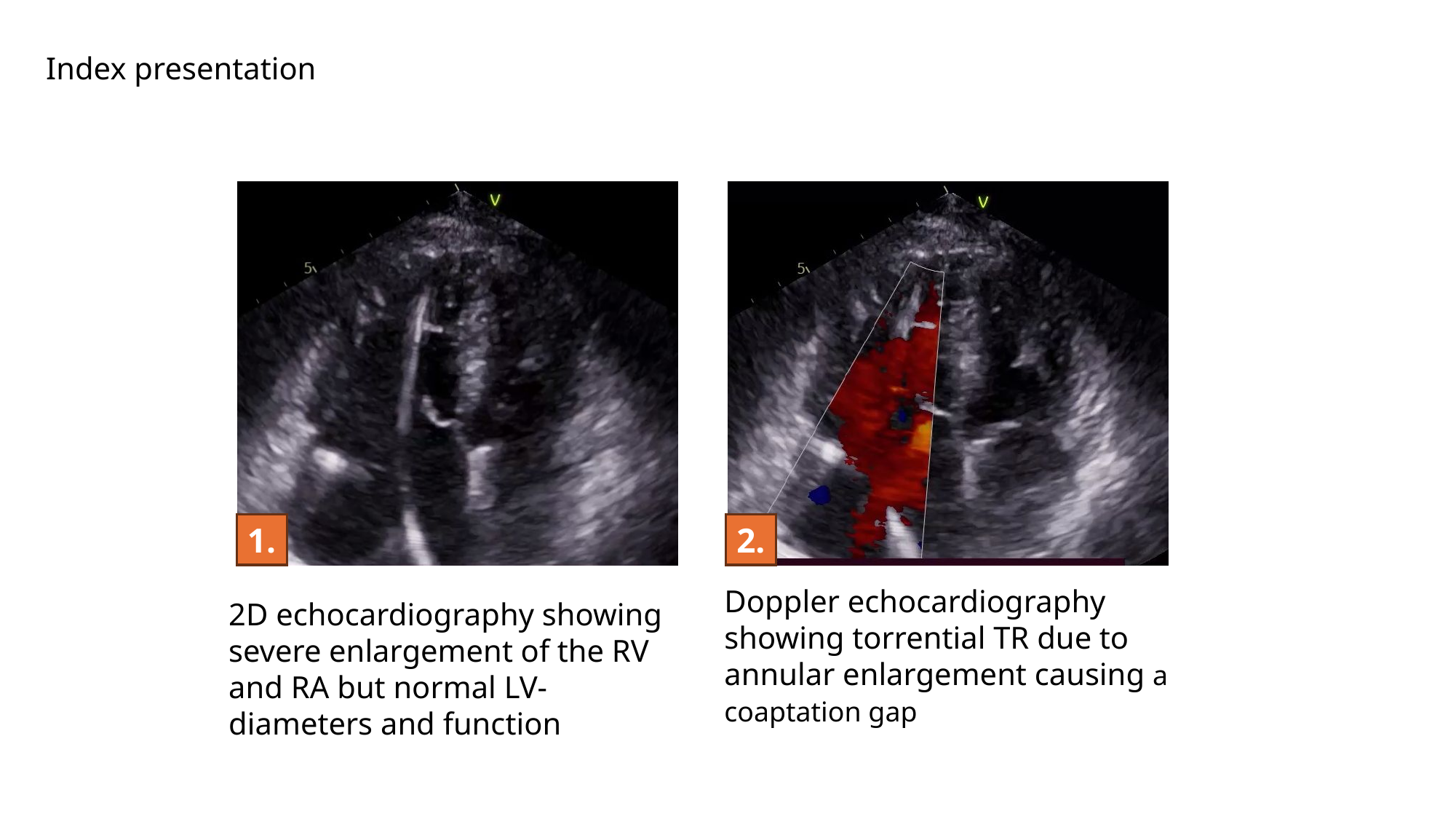

Index presentation
1.
2.
Doppler echocardiography showing torrential TR due to annular enlargement causing a coaptation gap
2D echocardiography showing severe enlargement of the RV and RA but normal LV-diameters and function

## Slide 3
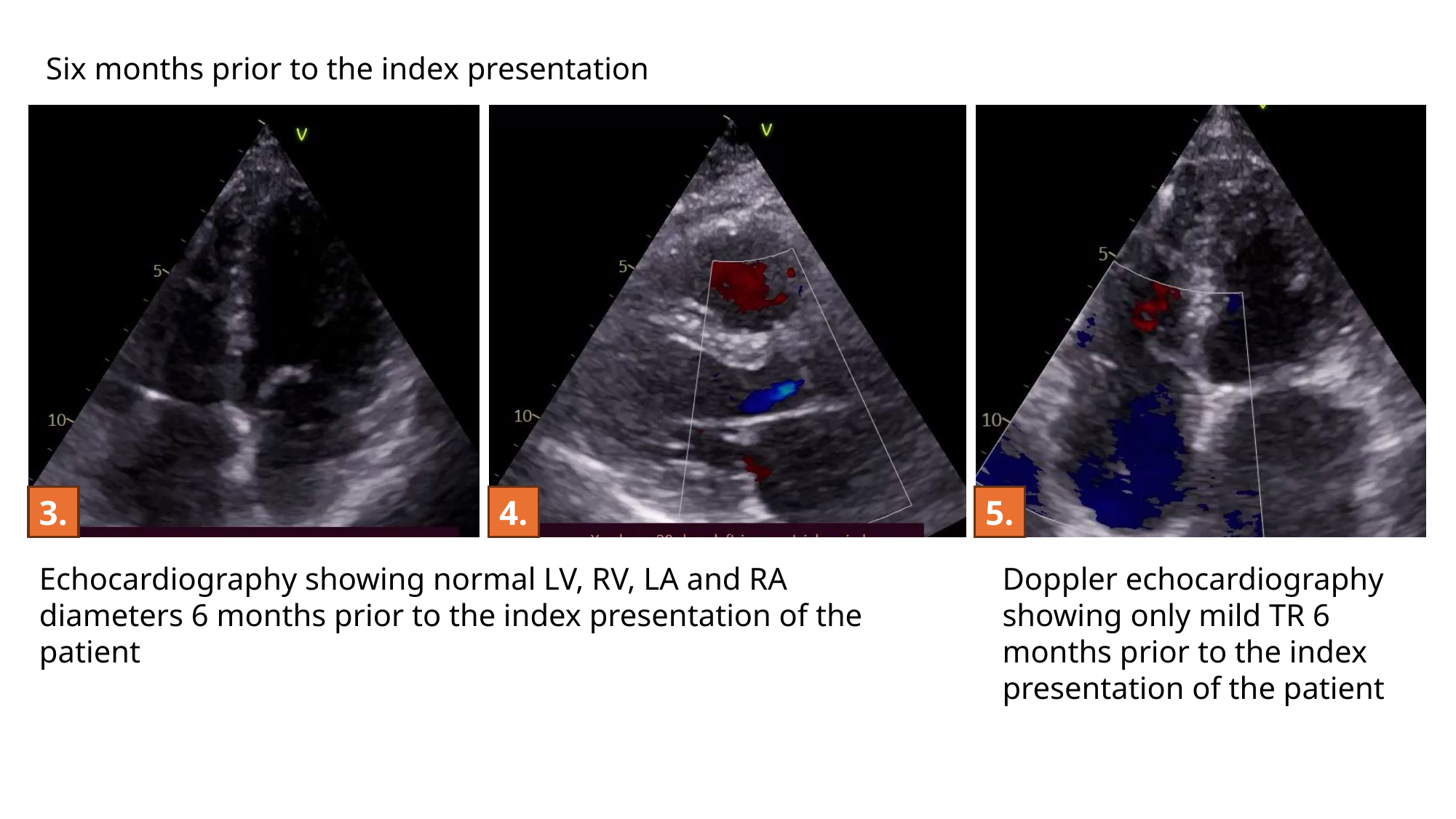

Six months prior to the index presentation
5.
4.
3.
Echocardiography showing normal LV, RV, LA and RA diameters 6 months prior to the index presentation of the patient
Doppler echocardiography showing only mild TR 6 months prior to the index presentation of the patient

## Slide 4
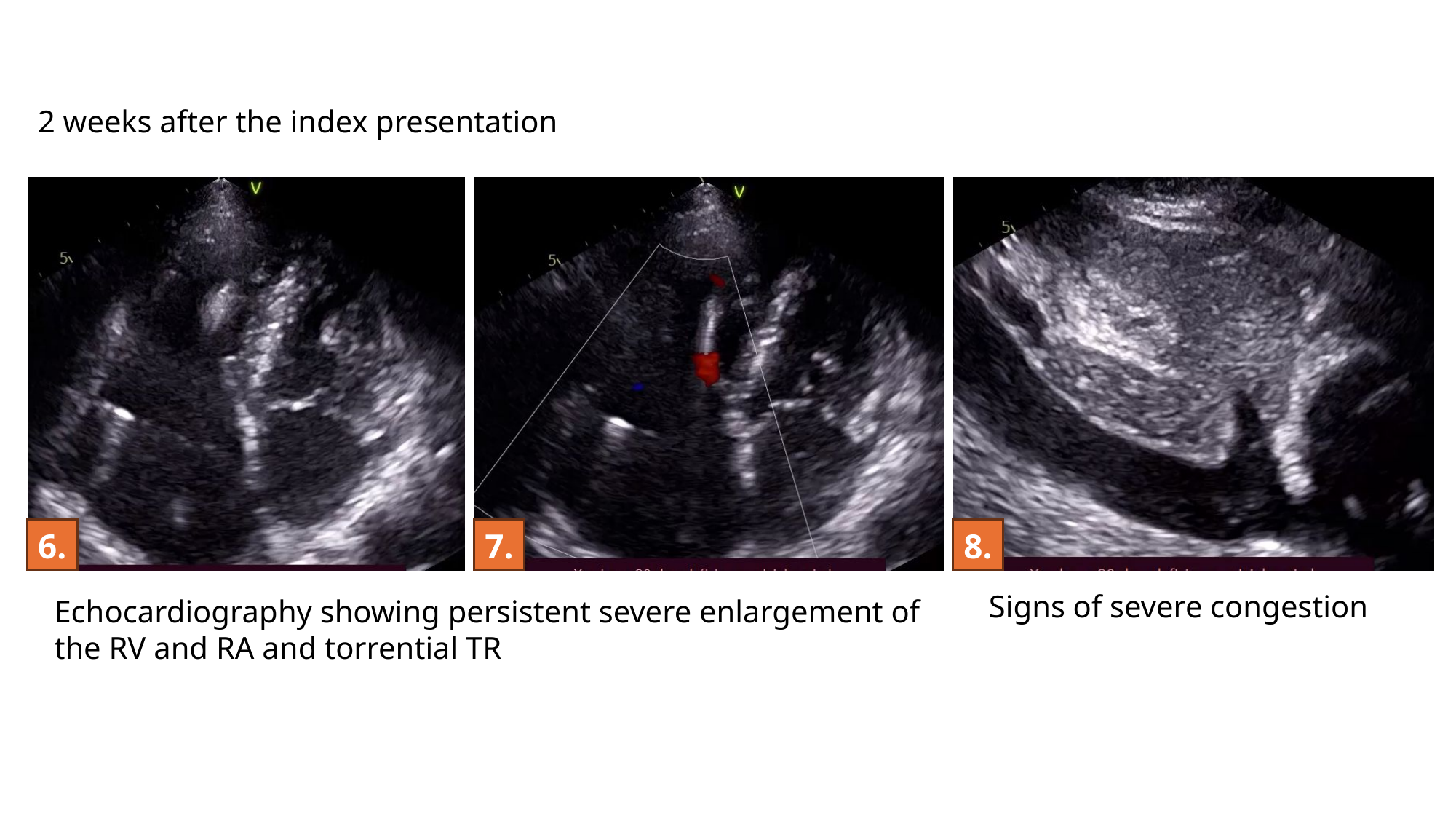

2 weeks after the index presentation
7.
8.
6.
Signs of severe congestion
Echocardiography showing persistent severe enlargement of the RV and RA and torrential TR

## Slide 5
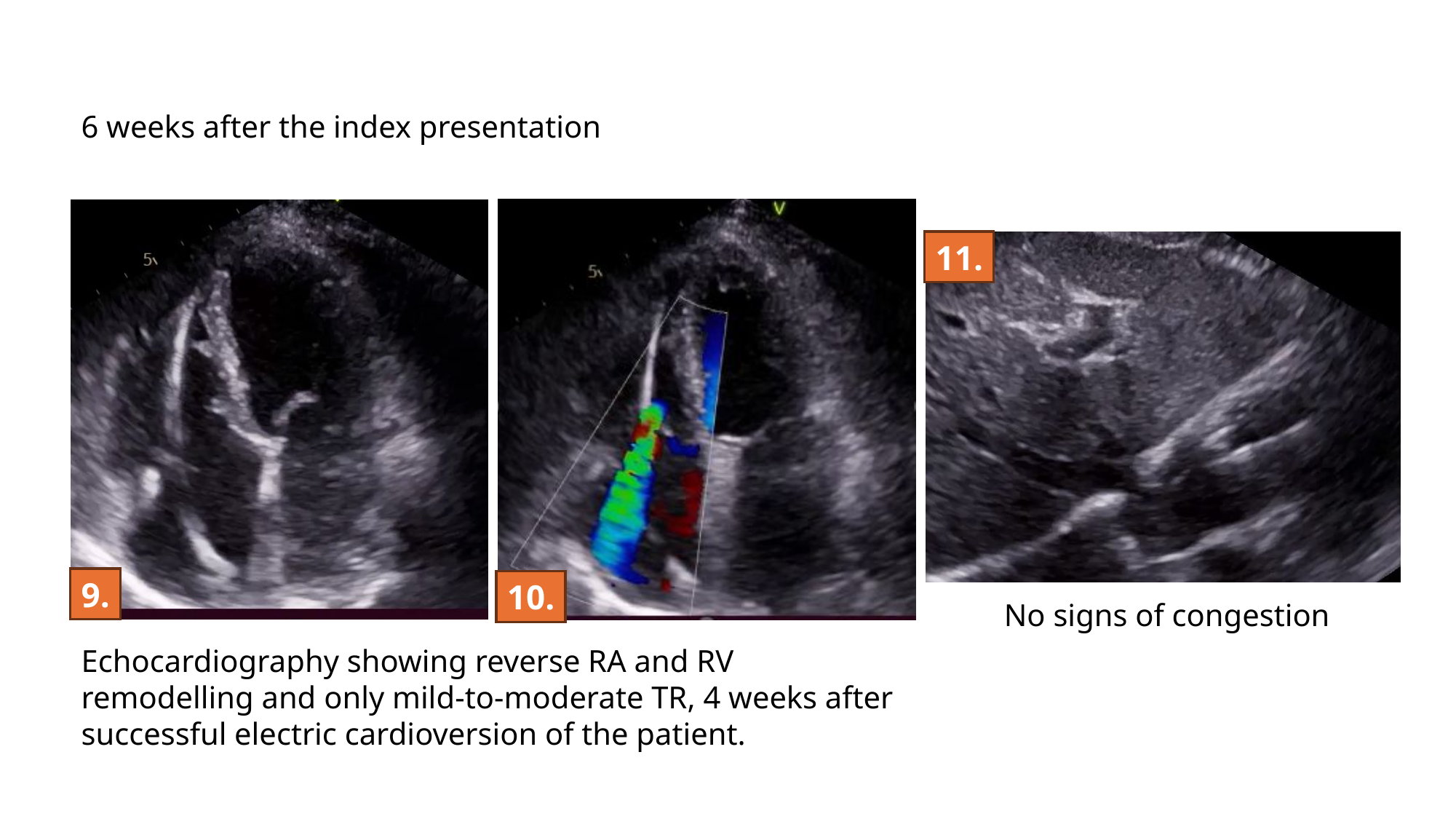

6 weeks after the index presentation
11.
9.
10.
No signs of congestion
Echocardiography showing reverse RA and RV remodelling and only mild-to-moderate TR, 4 weeks after successful electric cardioversion of the patient.

## Slide 6
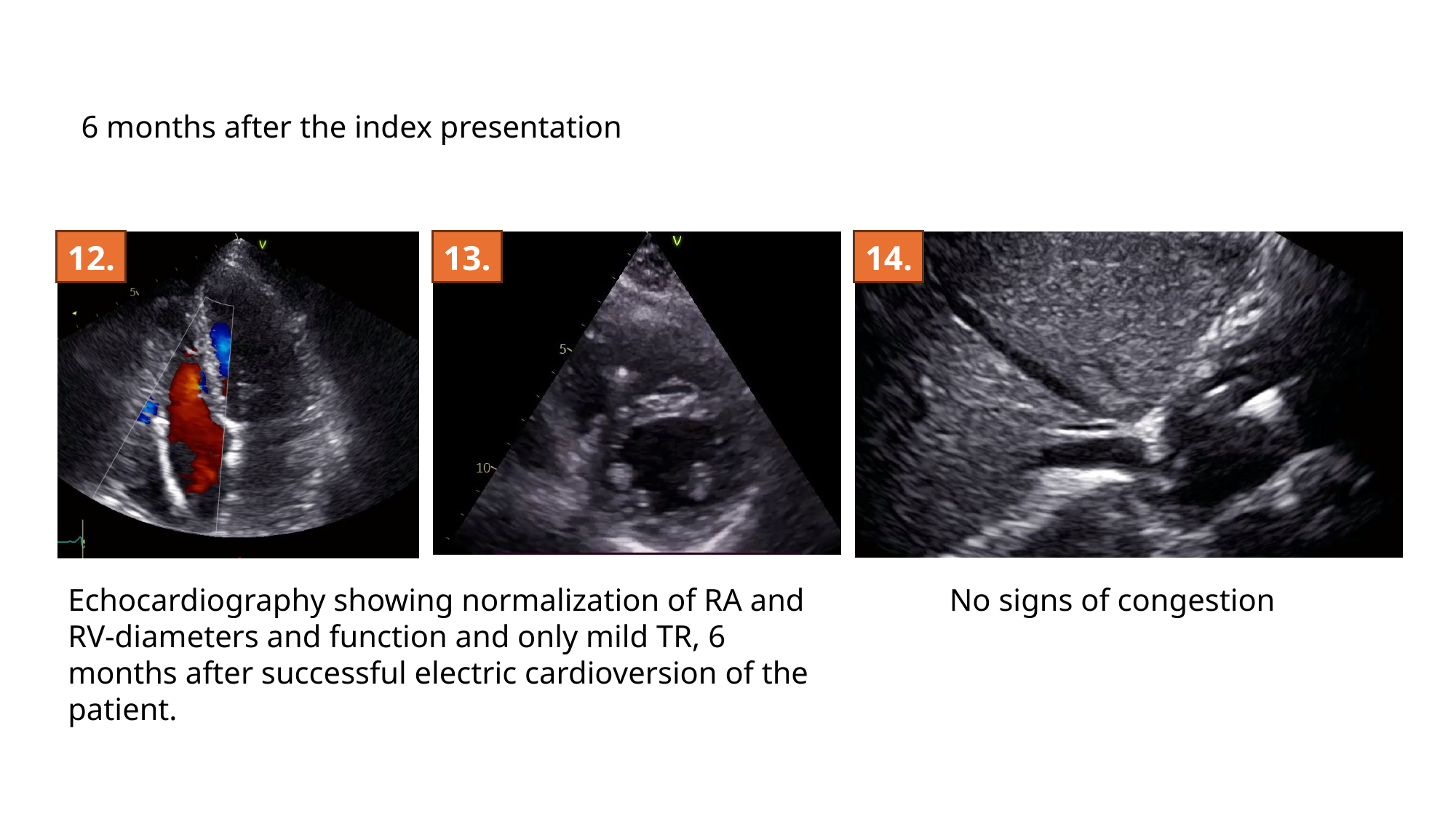

6 months after the index presentation
13.
14.
12.
Echocardiography showing normalization of RA and RV-diameters and function and only mild TR, 6 months after successful electric cardioversion of the patient.
No signs of congestion
